# The Synthesis of Europium-Doped Calcium Carbonate by an Eco-Method as Free Radical Generator Under Low-Intensity Ultrasonic Irradiation for Body Sculpture

**DOI:** 10.3389/fbioe.2021.765630

**Published:** 2021-11-19

**Authors:** Che-Yung Kuan, Yu-Ying Lin, I-Hsuan Yang, Ching-Yun Chen, Chih-Ying Chi, Chi-Han Li, Zhi-Yu Chen, Li-Ze Lin, Chun-Chen Yang, Feng-Huei Lin

**Affiliations:** ^1^ Institute of Biomedical Engineering, College of Medicine and College of Engineering, National Taiwan University, Taipei, Taiwan; ^2^ Institute of Biomedical Engineering and Nanomedicine, National Health Research Institutes, Miaoli County, Taiwan; ^3^ Ph.D. Program in Tissue Engineering and Regenerative Medicine, National Chung Hsing University, Taichung, Taiwan; ^4^ Department of Biomedical Sciences and Engineering, National Central University, Taoyuan, Taiwan; ^5^ Biomaterials Translational Research Center, China Medical University Hospital, Taichung, Taiwan; ^6^ Department of Materials Science and Engineering, National United University, Miaoli County, Taiwan; ^7^ Department of Materials Science and Engineering, National Taiwan University, Taipei, Taiwan

**Keywords:** calcium carbonate, europium, reactive oxygen species, body sculpture, ultrasound

## Abstract

Body sculpture is a common method to remove excessive fat. The diet and exercise are the first suggestion to keep body shape; however, those are difficult to keep adherence. Ultrasound has been developed for fat ablation; however, it could only serve as the side treatment along with liposuction. In the study, a sonosensitizer of europium-doped calcium carbonate (CaCO_3_: Eu) would be synthesized by an eco-method and combined with low-intensity ultrasound for lipolysis. The crystal structure of CaCO_3_: Eu was identified by x-ray diffractometer (XRD). The morphology of CaCO_3_: Eu was analyzed by scanning electron microscope (SEM). The chemical composition of CaCO_3_: Eu was evaluated by energy-dispersed spectrophotometer (EDS) and inductively coupled plasma mass spectrometer (ICP-MS). The electronic diffraction pattern was to further check crystal structure of the synthesized individual grain by transmission electron microscope (TEM). The particle size was determined by Zeta-sizer. Water-soluble tetrazolium salt (WST-1) were used to evaluate the cell viability. Chloromethyl-2′,7′-dichlorofluorescein diacetate (CM-H_2_DCFDA) and live/dead stain were used to evaluate feasibility *in vitro*. SD-rat was used to evaluate the safety and efficacy *in vivo*. The results showed that CaCO_3_: Eu had good biocompatibility and could produce reactive oxygen species (ROS) after treated with low-intensity ultrasound. After 4-weeks, the CaCO_3_: Eu exposed to ultrasound irradiation on SD rats could significantly decrease body weight, waistline, and subcutaneous adipose tissue. We believe that ROS from sonoluminescence, CO_2_-bomb and locally increasing Ca^2+^ level would be three major mechanisms to remove away adipo-tissue and inhibit adipogenesis. We could say that the combination of the CaCO_3_: Eu and low-intensity ultrasound would be a non-invasive treatment for the body sculpture.

## Introduction

The excessive localized fat is a matter of great concern among subjects from current society. It affects image and body shape negatively; and results in dissatisfaction on individual (de Gusmao et al., 2020). Body sculpture refers to the use of either surgical or non-invasive techniques to modify the body for those who desire to fat reduction for specific problem areas, such as, abdomen, hips, thighs (Jewell et al., 2012). In 2018, the global market to body sculpture reached to US$ 6.1 billion; that might increase to $16.5 billion by 2025 (Michon, 2021).

Generally, the diet and exercise are the first suggestion to keep body shape in normal or as so-called attraction (Kordi et al., 2015). However, strict diets and intense daily exercise are difficult to maintain routinely for much longer time; that may result to fail (Mason et al., 2018). Liposuction is a surgical technique used to remove fat tissue to make people have a desired contour, which is among the top five cosmetic surgical procedures performed in United States (Jalian and Avram, 2012). Unfortunately, the side effects of liposuction include lidocaine toxicity, infections, numbness, fat embolism, or even death. Furthermore, the skin may locally appear contour irregularities, for instance, bumpy, wavy or withered due to uneven fat removal, poor skin elasticity and unusual healing (Mrad et al., 2019; Witte et al., 2020; O’Neill et al., 2021). Along with safety concerns, several noninvasive nonsurgical approaches have been developed for body sculpting, which have drawn more attentions in recent years (Jalian and Avram, 2012; Shek et al., 2014).

Ultrasound is one of powerful tools in medical image for diagnosis and very popular in rehabilitation as a therapeutic modality (Moreno-Moraga et al., 2007). Over the last decade, ultrasound has been developed to a commercial set in plastic surgery as physical lipolysis for body sculpture by specific ultrasonic parameters to break down fat tissue around the patients’ waist.

As known, low-intensity ultrasound (0.5–17.5 W/cm^2^) would increase the inertial cavitation and then go through the bubble growth, finally to bubble implosion to generate the heat and stress to destroy the fat tissue for lipolysis (Zhou et al., 2017); however, the result of breaking down the fat tissue is not so promising, and it could only serve as the side treatment along with the liposuction (Tonucci et al., 2014). Alternatively, high-intensity focused ultrasound (HIFU) was developed to burn-down subcutaneous adipose tissue by high intensity (1,000 W/cm^2^) with a special focusing plate to converge the ultrasonic waves to the intended ablation area. HIFU has been reported to induce rapid cell necrosis by the high energy and temperature generated from cavitation explosion; that might effectively dissipate adipose tissue. However, HIFU has been reported to burn the surface skin and charred surrounding tissues, causing a serious inflammatory response.

In summary, lipolysis by low-intensity ultrasonic provides a good method for non-invasive and low-risk body sculpturing, without requiring a recovery period. However, non-invasive ultrasonic lipolysis still has some potential shortcomings that need to be improved and to skip the shortages from HIFU. In the study, a sonodynamic microparticles of europium-doped calcium carbonate would be synthesized to combine with low-intensity ultrasound for lipolysis on body sculpture by a mild and non-invasive way.

Calcium carbonate (CaCO_3_) is the candidate material selected for the study due to its excellent biocompatibility and stability (Xiao et al., 2021). CaCO_3_ is a biodegradable material that can decompose into carbon dioxide (CO_2_) and calcium ions (Ca^2+^) in the acidic environment of endosome-lysosome complex. It is also one of materials with the property of sonoluminescence; where the particle could absorb the energy from the explosion of ultrasonic cavitation to generate heat to react with oxygen or biomolecules to induce reactive oxygen species (ROS) generation, and then convert into different free radicals to de-nature the proteins for cell necrosis (Jonnalagadda et al., 2021). In addition, CO_2_ decomposed from CaCO_3_ may serve as bomb to make cell damage under explosive stress, that could further kill the adipocyte (Yang et al., 2019). Ca^2+^ released from the breaking down of CaCO_3_ at the acidic endosome-lysosome complex would increase the local calcium level around the adipose tissue; that might inhibit the differentiation of mesenchymal stem cells toward adipogenesis (Li et al., 2018). We believe that ROS from sonoluminescence, CO_2_-bomb and locally increasing Ca^2+^ level would be three major mechanisms to effectively remove away adipo-tissue for body sculpture.

In order to increase the sonoluminescent effect, a rare element Eu, would be doped into the crystal lattice to partially replace the Ca^2+^ in the lattice site of CaCO_3_. A green method was developed to synthesize the particle of Eu-doped CaCO_3_ (CaCO_3_: Eu) at relative-lower temperature without organic solvent involved in.

In this study, x-ray diffractometer (XRD) was used for the crystal structure identification of the synthesized CaCO_3_: Eu. The morphology of the developed particle was observed by scanning electron microscope (SEM). The semi-quantitative chemical composition of the developed particle was examined and evaluated by energy-dispersed spectrophotometer (EDS) and inductively coupled plasma mass spectrometer (ICP-MS). The electronic diffraction pattern was to further check the crystal structure of the synthesized individual grain by transmission electron microscope (TEM). The particle size was determined using a Zeta-sizer. The water-soluble tetrazolium salt (WST-1) on L-929 cells were used to evaluate the cell viability of the developed material; that would be in terms of *in vitro* cytotoxicity. Chloromethyl-2′,7′-dichlorofluorescein diacetate (CM-H_2_DCFDA) and live/dead stain were used to evaluate how the combination of CaCO_3_: Eu and low-intensity ultrasound works on 3T3-L1; the results would serve as first screening *in vitro*. Finally, SD-rat was used as the target animal to evaluate the safety and efficacy *in vivo*; where the body weight, body temperature, waist line, the weight of subcutaneous adipose tissue on ultrasonic area, histological sectioning, blood element analysis, and serological analysis would be measured and checked to prove the concept.

**FIGURE 1:**
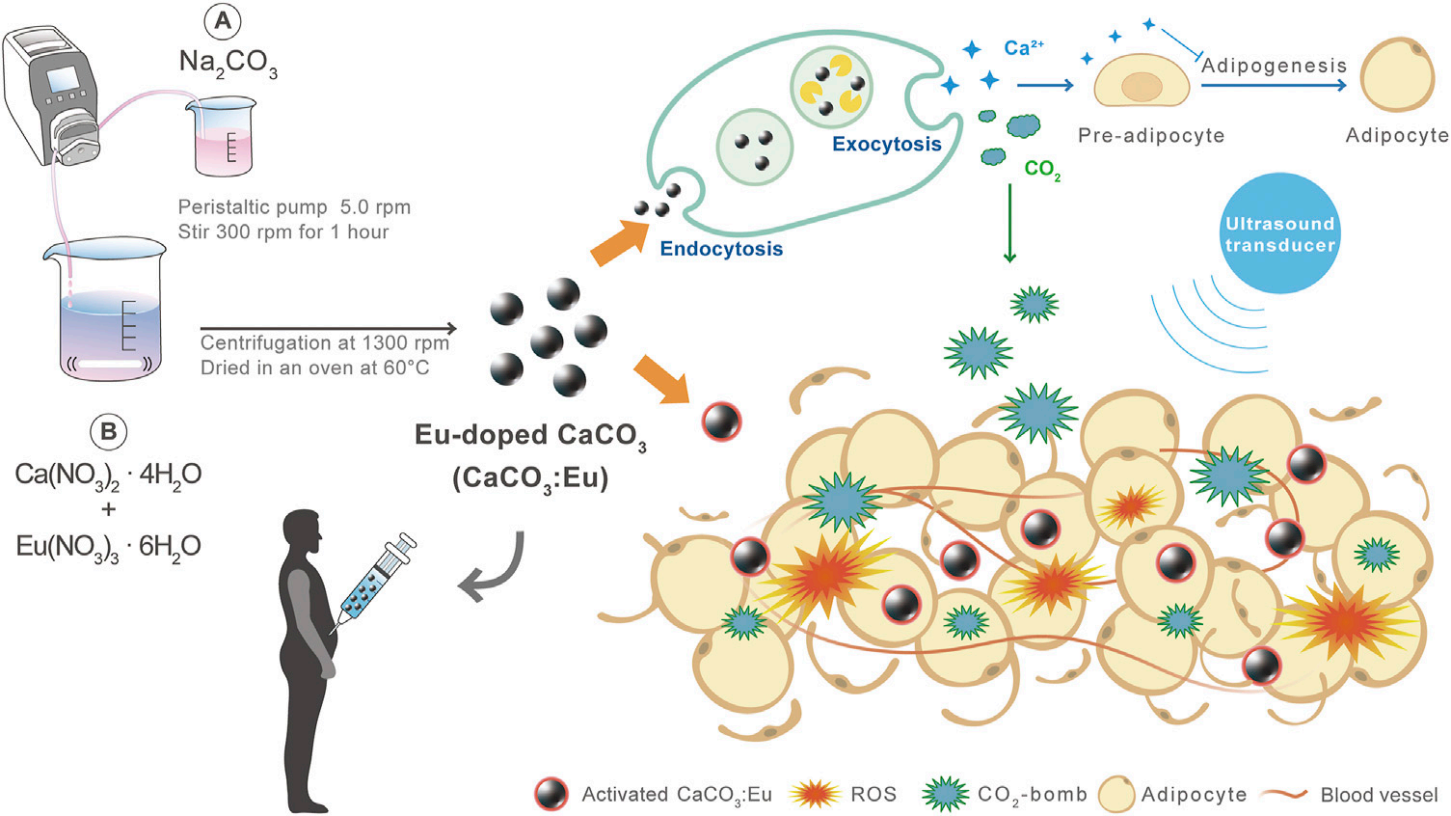
The major mechanisms of CaCO_3_: Eu with low-intensity ultrasound treatment to effectively remove away adipo-tissue and inhibit adipogenesis for body sculpture.

The scenario of the study was firstly to synthesize a high-sonoluminescent CaCO_3_: Eu particles by a new developed method. Secondly, the synthesized particles would be injected to abdomen area and then locally applied with low-intensity ultrasound to prevent from the skin burning and charred surrounding tissue. The combination of sonoluminescent CaCO_3_: Eu and low-power ultrasound would generate ROS to damage the adipo-tissue under the stress of free radicals. The CO_2_ and Ca^2+^ decomposed from CaCO_3_: Eu would serve as CO_2_-bomb and increase local Ca^2+^ level to further break-down the adipo-tissue and to inhibit local adipogenesis. The overall process would be schemed in Figure 1.

## Materials and Methods

### Europium-Doped Calcium Carbonate Preparation

CaCO_3_: Eu was synthesized by an innovative method at room temperature without organic solvent addition for environment friendly. The process was briefly described as follows. Firstly, 1.18 g of calcium nitrate and 0.223 g of europium nitrate was dissolved in 50 ml of ddH_2_O. Then, 50 ml of 0.1 M sodium carbonate was added drop-by-drop into the previously prepared calcium/europium nitrate solution by peristaltic pump at 5.0 rpm and stirred by magnetic stirrer at room temperature for 3 h, and the solution was centrifugated at 1,300 rpm for 20 min (5500, Kubota, Japan). The precipitate was washed by ddH_2_O for three times, and dried overnight in a freeze dryer (FDU-1100, EYELA, Japan) to obtain CaCO_3_: Eu. The synthesized particles were stored in desiccator for later use.

### The Crystal Structure Identification

The crystal structure of the synthesized particles was identified by XRD (MiniFlex II, Rigaku, Japan) with Copper Kα-II radiation at 30 kV and 15 mA at a scan rate of 4°/min from 20 to 60°. The sample was passed 230 mesh and pressed onto a sample holder with an area of 2 cm × 2 cm.

### The Morphological Examination and Grain Size Evaluation Under SEM

The morphology and grain size of the synthesized particles were examined and observed by a SEM (Hitachi TM-1000, Japan). The sample were mounted on an aluminum-made SEM sample stage and then coated with a platinum film by a sputtering PVD. The sample edge was spotted with silver gel to prevent from undesired discharge to result in a blurry image.

### The Analysis of Morphology and Electronic Diffraction Pattern by TEM

The morphology and electronic diffraction pattern of the developed particles were observed and analyzed by TEM (Tecnai G2 F20, FEI, United States). 5 mg of the particles were dispersed in 10 ml ddH_2_O and homogenized by ultrasonic vibration for 15 min 20 μl of the dispersed and homogenized particles were dropped on the carbon-coated copper mesh, and dried at room temperature in a petri-dish with lid covered to prevent from pollution from air. The accelerated voltage was 200 kV. The electronic diffraction pattern was obtained by selected area diffraction mode (SAD-mode).

### Chemical Composition Analysis

The chemical composition of the material was analyzed by an EDS (JSM-5600, JEOL, Japan). The sample preparation was similar to process of the sample for SEM, but coated with a pyrolytic carbon rather than platinum film. The energy of the accelerated x-ray beam was 20 kV. The chemical composition of sample was further confirmed by an inductively coupled plasma mass spectrometer (ICP-MS, NexION 2000, PerkinElmer, United States). In brief, 20 mg of sample was dissolved in 200 μl of pure nitric acid (438073, Sigma, United States), and added with ddH_2_O to 10 ml. The simple was diluted (1:10,000) with ddH_2_O and performed by ICP-MS with kinetic energy discrimination (KED) mode.

### The Analysis of Particle Size Distribution

The particle size distribution of the synthesized particles was analyzed by using a Zeta-sizer (Nano ZS, Malvern, United Kingdom). The sample was firstly suspended in ddH_2_O and homogenized by an ultrasonic vibration. The homogenized suspension was placed in a Zeta-sizer cell and then measured using Dynamic Light Scattering (DLS) at room temperature.

### *In Vitro* Study

#### Evaluation of Cell Viability

The cell viability was evaluated by WST-1 on L-929 cell (RM60091, Bioresource Collection and Research Center, Taiwan); that would be in terms of *in-vitro* cytotoxicity based on the guideline of ISO 10993-5.

Briefly, L-929 cells were cultured in α-MEM (11900-024, Gibco, United States) supplemented with 10% fetal bovine serum (FBS, A31606-02, Hyclone, United States) and 1% of 100X antibiotic-antimycotic (Anti-anti, 15240-062, Gibco, United States); and then seeded to a 96-well culture plate with a cell density of 1 × 10^4^ per well and cultured at 37°C under 5% CO_2_ for 24 h.

The culture medium would be used as the extraction vehicle to prepare sample extracted solution. 0.2 g of developed particles, aluminum oxide (11028, Sigma, United States) and polyurethane film containing 0.1% zinc diethyldithiocarbamate (ZDEC, RM-A, Hatano Research Institute, Food and Drug Safety Center, Japan) were immersed in 1 ml of culture medium, individually, at 37°C under 5% CO_2_ for 24 h. The extracted solutions would be separately cultured with previous seeded cells and daily refreshed to evaluate cell viability; those would be named and abbreviated as experimental group (CaCO_3_: Eu), negative control (N-control) and positive control (P-control), respectively. The result of L-929 cells cultured with medium were the control group abbreviated as Control.

After 1-day incubation, the medium was removed and then added in 90 μl culture medium and 10 μl WST-1 reagent (11644807001, Roche, United States); that was reacted at 37°C under 5% CO_2_ for 1 h in dark. The culture plate was mounted on ELISA reader (VersaMax™, Molecular Devices, Canada); where the absorbance at the wavelength of 450 nm was recorded to evaluate the cell viability (Hsiao et al., 2019).

#### 3T3-L1 Culture and Differentiation

Briefly, 3T3-L1 pre-adipocytes cell line (60159, Bioresource Collection and Research Center, Taiwan) was seeded to a 12-well culture plate with a cell density of 1 × 10^4^ per well and cultured at 37°C under 5% CO_2_ in Dulbecco Modified Eagle Medium (DMEM, high glucose, 12800-017, Gibco, United States) supplemented with 10% calf bovine serum (16170-078, Gibco, United States) and 1% of 100X Anti-anti. After confluence, it were further cultured in starvation condition for 2 days to keep cells in the status of G_0_/G_1_ phase at least 85% in all population (Cao et al., 2012). The confluent 3T3-L1 cells were cultured in an adipo-differentiated medium to convert cells into adipocytes; where the adipo-differentiated medium was DMEM supplemented with 10% FBS, 1% of 100X Anti-anti, 1 mM dexamethasone (D4902, Sigma, United States), 0.2 M indomethacin (I7378, Sigma, United States), 0.1% insulin and 0.25 M 3-Isobutyl-1-methylxanthine (IBMX, I5879, Sigma, United States). The adipocytes were cultured in DMEM supplemented 10% FBS and 1% of 100X Anti-anti; and medium was refreshed every 3 days, until the oil droplets were observed by a fluorescence microscope (TS-100, Nikon, Japan) stained with Nile red (N1142, Invitrogen, United States) (Park et al., 2017).

#### ROS Generation

The ROS generation of adipocytes, induced by synthesized CaCO_3_: Eu and exposed to low-intensity ultrasound, was measured by CM-H_2_DCFDA (C6827, Invitrogen, United States).

In brief, 3T3-L1 cells were seeded into 96-well culture plate with a density of 1 × 10^4^ cells per well and differentiated to adipocyte as described in *3T3-L1 Culture and Differentiation*. 100 μl of 0.75 mg/ml CaCO_3_: Eu in culture medium was added into each well and further cultured for 4 h, and then exposed to low-intensity ultrasound from the bottom of the culture plate in degassed water by an ultrasound transducer with a diameter of 2.0 cm. The distance between ultrasound transducer and the bottom of the cell culture plate was around 5 mm. The ultrasound irradiation was conducted with a function generator (33521A, Agilent, United States) at a resonant frequency of 1.0 MHz and a duty cycle of 50%. A power amplify was used to generate a square wave with a negative pressure of 0.33 MPa and intensity of 1.8 W/cm^2^ for 90 s (Yang et al., 2020). It was further cultured for 1 h in the incubator. The medium was removed and the cells were stained with 25 μM CM-H_2_DCFDA at room temperature for 45 min. The fluorescence was excited at the wavelength of 493 nm; and the intensity of emission light was measured by a multi-label plate reader (EnSpire, PerkinElmer, United States) at the wavelength of 523 nm that was the ROS concentration.

The experiment was divided into four groups and abbreviated in brace as follows: the cells were cultured in medium, 1) without CaCO_3_: Eu addition and no ultrasound applied on (Control); 2) applied with low-intensity ultrasound without CaCO_3_: Eu addition (US); 3) with CaCO_3_: Eu addition but no expose to low-intensity ultrasound (CaCO_3_: Eu); 4) with CaCO_3_: Eu addition and expose to low-intensity ultrasound (US-CaCO_3_: Eu).

#### The *In Vitro* Screening of Adipocyte Treated With Synthesized CaCO_3_: Eu and Low-Intensity Ultrasound by WST-1 Assay and Live/Dead Stain

The cell viability and cytotoxicity of adipocyte, treated with synthesized CaCO_3_: Eu and exposed to low-intensity ultrasound, were evaluated by WST-1 assay and live/dead stain, respectively. The experiments were used as first screening *in-vitro,* trying to know the possibility of body sculpture *in vivo* once adipo-tissue treated with developed particles and followed by low-intensity ultrasound irradiation.

In brief, 3T3-L1 cells were seeded on 12-well culture plate with a density of 6 × 10^4^ cells per well and then differentiated into adipocyte. 0.75 mg/ml CaCO_3_: Eu was added into each well and further cultured for 4 h, and then exposed to low-intensity ultrasound. It was further cultured for 1 h in the incubator. The medium was removed and then added in 900 μl culture medium and 100 μl WST-1 reagent; that was reacted at 37°C under 5% CO_2_ for 1 h in dark. The culture plate was mounted on ELISA reader (EnSpire, PerkinElmer, United States); where the absorbance at the wavelength of 450 nm was recorded to evaluate the cell viability.

In the live/dead staining, the staining solution was prepared as follows; in which 50 μl of calcein AM (Ex/Em: 494/517 nm, C1430, Invitrogen, United States) and 16.5 μl of propidium iodide (PI, Ex/Em: 536/617 nm, P1304MP, Invitrogen, United States) reagents were well-mixed in phosphate buffered saline (PBS) and then added PBS to 5 ml, at pH 7.4. As previous description, the adipocytes were treated by developed CaCO_3_: Eu and low-intensity ultrasound. After further cultured for 1 h, the medium was removed and added in 400 μl of staining solution, reacted for 15 min at room temperature in dark. The culture plate was mounted on fluorescence microscope (TS100, Nikon, Japan), with which the living cells and dead cells would be labelled by calcein AM in green color and propidium iodide in red, respectively, under the proper excitation light.

### *In Vivo* Study

#### Experimental Animals and Surgical Procedure

Sprague Dawley rat age 10-weeks old, 325 g body weight in average and male in gender was used in the study. The rats were purchased from BioLASCO, Taiwan, and delivered to Laboratory Animal Center, National Health Research Institutes, Taiwan, 7 days before the experiment started to accommodate the environment. One cage for one rat was conducted to all the experimental period with controlled temperature and humidity of 22°C and 55%, respectively, by light turn-off and turn-on alternatively every 12 h. The study protocol was approved by the Institutional Animal Care and Use Committee of the National Health Research Institutes (NHRI-IACUC-108012).

3.75 g of CaCO_3_: Eu was mixed within 1 ml of normal saline. The 100 μl of mixture was injected into the fat tissue of abdomen area on the SD rats once a week for 4 weeks. The low-intensity ultrasound was applied on the area where CaCO_3_: Eu was injected; and treated consecutively 3 days every week for 4 weeks, each day 90 s. The low-intensity ultrasound was generated by a function generator at a resonant frequency of 1.0 MHz, a duty cycle of 50%, a square wave with a negative pressure of 0.33 MPa and intensity of 1.8 W/cm^2^.

The study was divided into three groups that was described and abbreviated as follows: 1) the rats without any treatment were categorized to Control Group (Control); 2) the rat received injection on abdomen fat tissue once a week by 100 μl normal saline was Sham Control; 3) the rat injected with CaCO_3_: Eu once a week and received ultrasound treatment consecutively 3 days every week was the major experimental group, abbreviated as US- CaCO_3_: Eu.

The body weight, body temperature, weight and waistline of the experimental rats were measured and recorded every week. At the end of the experiment, the rats were sacrificed and the blood was collected directly from the heart. The subcutaneous fat and organs were harvested for further analysis.

#### Serological and Blood Elements Analysis

In the serum analysis, the blood was collected in a blood collection tube (450533, Greiner bio-one, Austria), and centrifuged at 3,500 rpm for 10 min in a centrifuge (5500, Kubota, Japan). The supernatant was collected and analyzed. Blood lipid (TC, TG), liver function (AST, ALT), renal function (BUN, Creatinine, UA), and calcium (Ca) were analyzed by serology analyzer (DRI/CHEN NX-500 I, Fuji, Japan).

In the blood elements, the blood was collected in a purple collection tube containing an EDTA anticoagulant, and mixed homogeneously for analysis. The number of white blood cells (WBC), red blood cells (RBC), hemoglobin (HGB), hematocrit ratio (HCT), platelets (PLT), neutrophil (NE), eosinophilic multinuclear (EO), basophil (BA), lymphocytes (LY), and mononuclear spheres (MO) were analyzed by hematology analyzer (BC-5000 VET, Mindray, China).

The two analysis were to check the safety of the new developed lipolysis method on the experimental animal. The results were recorded and summarized in the Supplementary Data Sheet S1.

#### Histological Sectioning With Hematoxylin and Eosin Stain

The tissue sample of heart, liver, spleen, lungs, and kidneys were harvested by a sterilized surgical instrument. The tissues were carefully trimmed the surroundings and cleaned by PBS; and then placed in a 10% formalin solution (HT501128, Sigma, United States) for fixation. It was then immersed in acetone to de-oil and dehydrated by series of alcohol from 70 to 100%. The tissue was paraffin embedding in a tissue embedder (TEC-6, Tissue-Tek, United States). The paraffin blocks were sectioned (5 mm thick sections) on a rotary microtome (RM 215, Leica, Germany), and then the sections were fixed in 4% paraformaldehyde for 20 min, and washed 2 times by ddH_2_O for 30 s. Dipped the slides into a Coplin jar containing hematoxylin solution for 30 s. Rinsed slides by ddH_2_O for 1 min, and then stained with 1% eosin Y solution for 20 s. Dehydrated the sections with 2 times by 95% alcohol and two changed of 100% alcohol. The sections were cleaned by xylene for 5 min and put on cover slide by mounting media (Zihayat et al., 2018). The images were observation by an optical microscope (Eclipse 80i, Nikon, Japan). The results were summarized in the Supplementary Data Sheet S1.

### Statistic Method

All the experiments were conducted at least in triplicate, and the data was presented with means ± SD. Statistical analyses were performed by one-way ANOVA. The results were considered significant difference when the *p*-value < 0.05.

## Results

### Material Characterization

#### The Crystal Structure Identification

**FIGURE 2:**
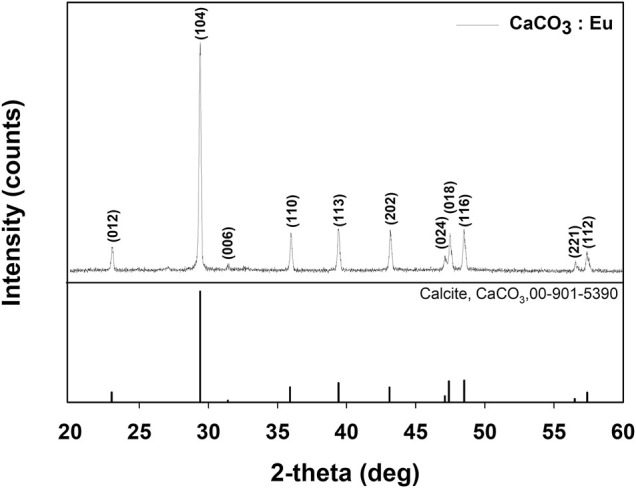
XRD pattern of CaCO_3_: Eu.

Figure 2 showed XRD patterns of the synthesized CaCO_3_: Eu. The characteristic peaks appeared at *2θ* of 23.0°, 29.4°, 31.4°, 35.9°, 39.4°, 43.1°, 47.1°, 47.4°, 48.5°,56.5°, and 57.4 were corresponding to the plane of (012), (104), (006), (110), (113), (202), (024), (018), (116), (221), and (112), respectively. The peaks and relative intensities of the synthesized CaCO_3_: Eu were fully matched to the calcite CaCO_3_ as Crystallography Open Database (COD) No. 00-901-5390.

**FIGURE 3:**
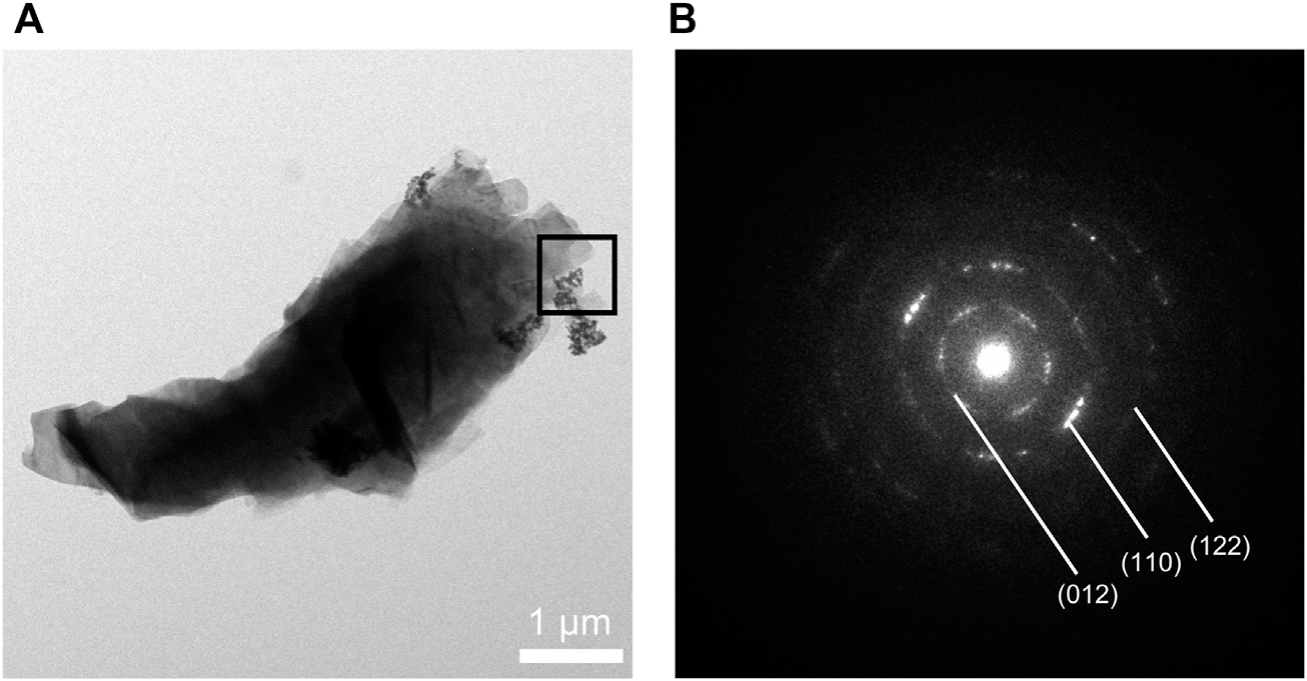
**(A)** TEM photo of CaCO_3_: Eu, **(B)** selected area electronic diffraction pattern of CaCO_3_: Eu.

The synthesized CaCO_3_: Eu further examined under the TEM; that showed a “nailhead” or “dogtooth” spar of calcite crystals that grew and aggregated with different habits, as shown in the edge in upper right of the Figure 3A. The selected electronic diffraction pattern (Figure 3B) was a classic ring pattern; with which the d-spacings calculated from the ring pattern were in agreement with the plane of (012), (110), and (122) in calcite crystal structure coded with COD No. 00-901-5390.

#### The Morphological Examination and Grain Size Evaluation Under SEM

**FIGURE 4:**
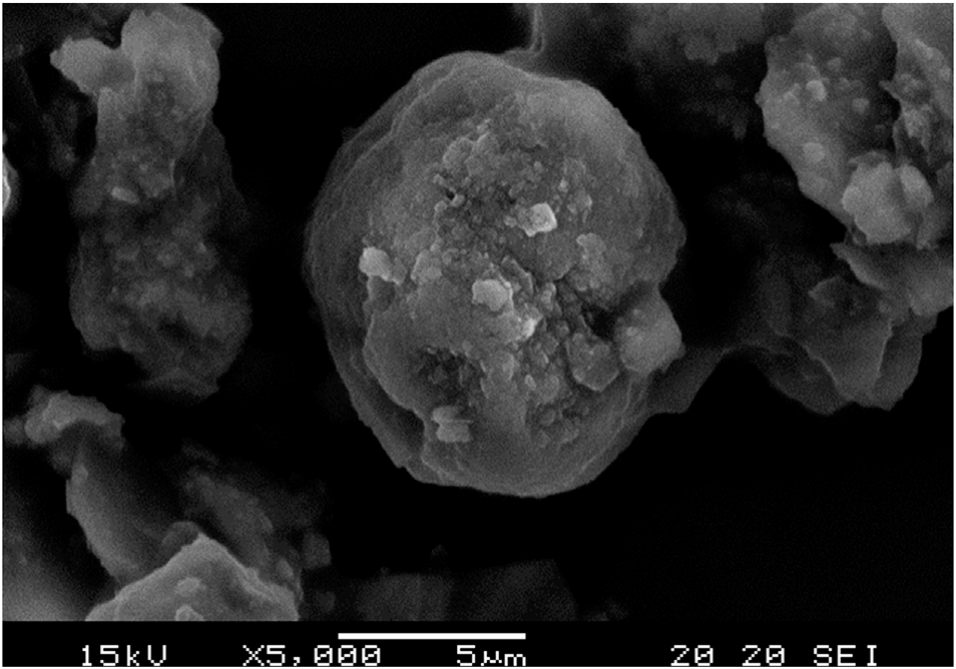
SEM image of CaCO_3_: Eu.

The surface morphologies of the developed CaCO_3_: Eu were examined under SEM as shown in Figure 4. It was aggregated into a particle approximately 4 μm in average; that was composed by many small rhombohedral grains stacking into a particle. The particle was shaped as scalenohedron or prism by the nano-sized grains; that could be seen from the edge of TEM photo as Figure 3A.

#### Chemical Composition Analysis

**FIGURE 5:**
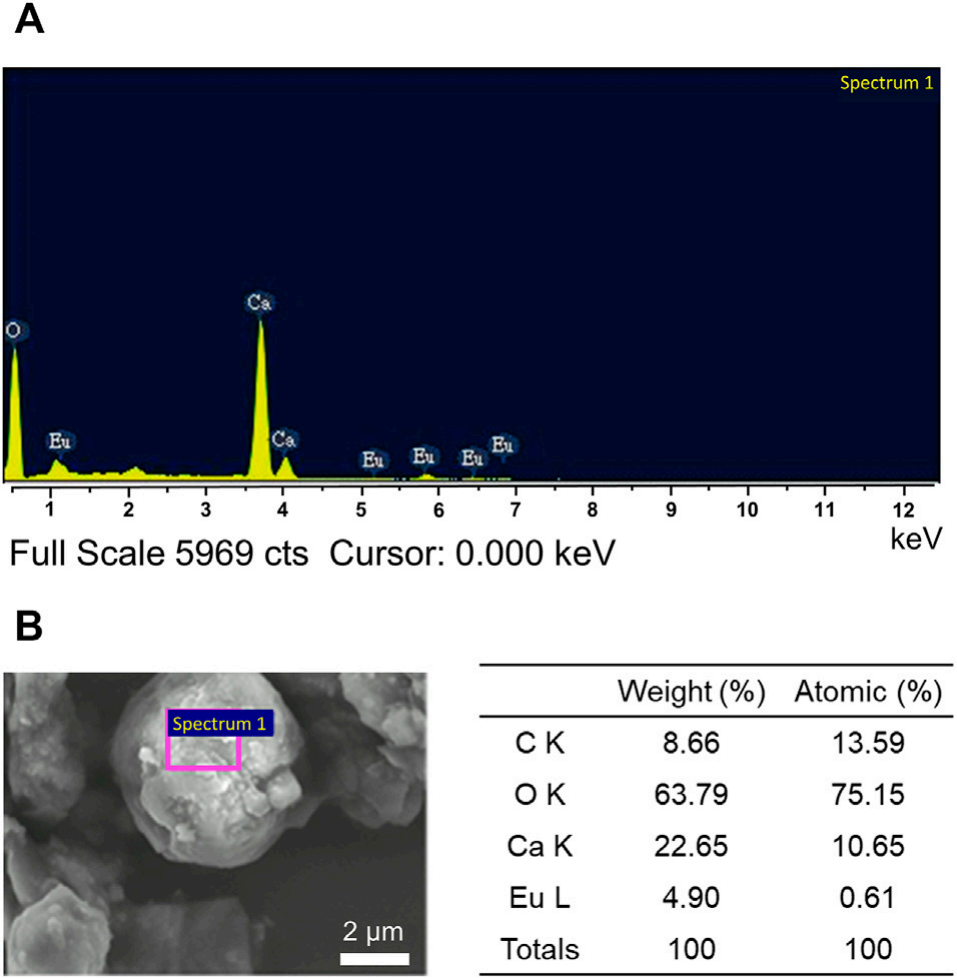
**(A)** The chemical composition of the synthesized CaCO_3_: Eu by EDS, and **(B)** the weight percentage and atomic percentage in average of each element.

The overall elements composed in the synthesized CaCO_3_: Eu was detected by energy dispersed spectrophotometry to analyze the energy status of the electrons in different orbits as shown in Figure 5A; where the major elements were carbon, oxygen, calcium and europium. The average weight percentage (weight%) and average atomic percentage (atomic%) of each element were shown in Figure 5B. An ICP-MS was used to further confirm the concentration of Eu in synthesized particle. The concentration of Eu in CaCO_3_: Eu was 112.5 mg/g (Supplementary Table S1). In this study, the molar ratio of europium to calcium in the CaCO_3_: Eu was 0.084; that was high substitution rate of Eu to Ca in the calcite lattice site as 8.4% due to similar atomic radius and valence.

#### The Analysis of Particle Size Distribution

**FIGURE 6:**
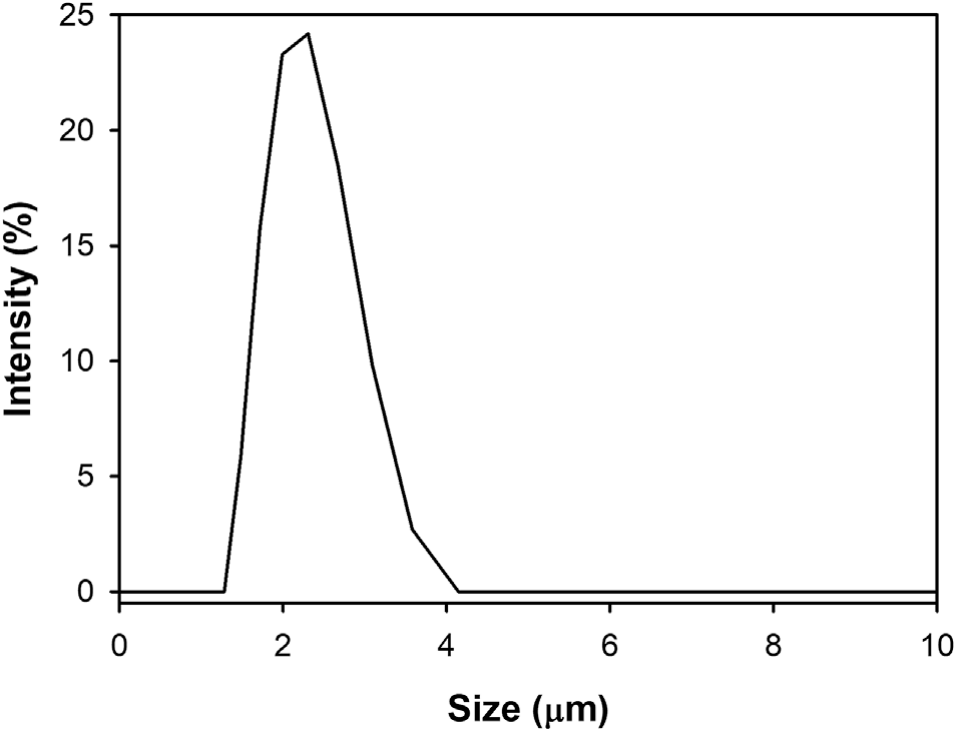
Size distribution of CaCO_3_: Eu.

A Zeta-sizer was used to analyze the particle size and distribution of the synthesized CaCO_3_: Eu. As shown in Figure 6, the particle size of CaCO_3_: Eu was approximately 2.1 μm in average, and the size distribution of CaCO_3_: Eu is from 1.48 to 3.58 μm; that was very close to the 4 μm in average observed under SEM. In the SEM picture (Figure 4), the particle might more aggregate into a bigger one during drying process in the sample preparation. We believe that the developed CaCO_3_: Eu with adequate particle size could be uptake by the defense cells, such as phagocyte, macrophage etc., for the later-on controlled release by endosome-lysosome complex breaking down and pumping out to extra-cellular matrix, finally delivered to whole body by the surrounding capillary system. We would prove it in the later experiments.

### Evaluation of Cytotoxicity *In Vitro*

**FIGURE 7:**
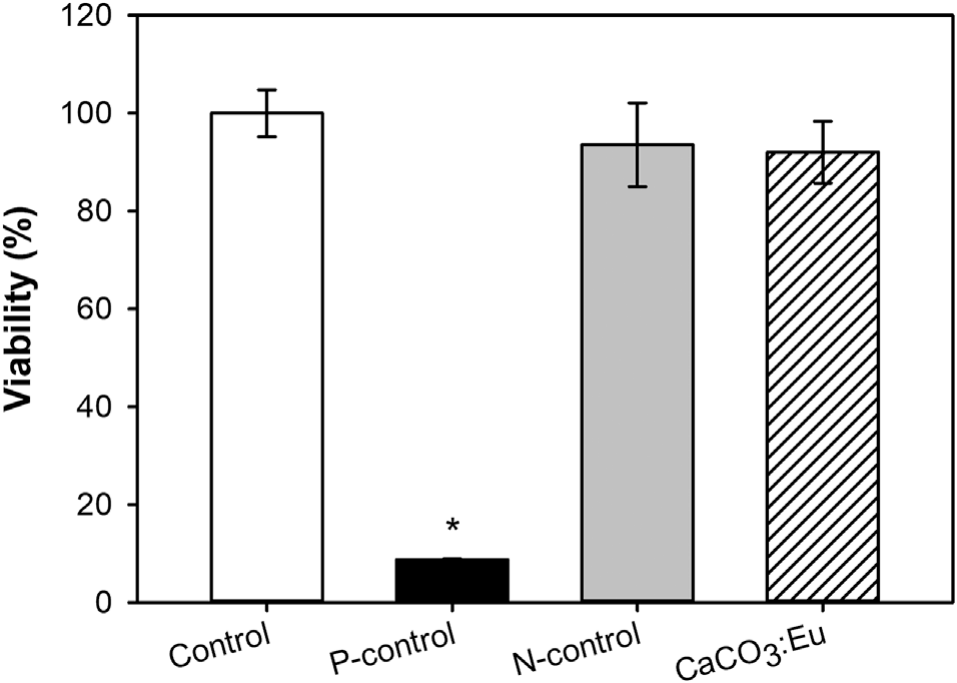
The evaluation of cell viability of synthesized CaCO_3_: Eu. *p < 0.05.

Figure 7 showed the cell viability of the developed CaCO_3_: Eu followed the guideline of ISO 10993-5. The cell viability of the control group, P-control, N-control and experimental group of CaCO_3_: Eu were 100 ± 4.82, 8.70 ± 0.19, 93.57 ± 8.54, and 92.05 ± 6.293, respectively. The difference of OD value between control group and CaCO_3_: Eu was less than 25%. We could tell that the synthesized CaCO_3_: Eu would not induce cytotoxicity to L-929 cells; and would keep cellular metabolism and mitochondrial functions in normal.

### ROS Generation of CaCO_3_: Eu Expose to Ultrasonic Irradiation

**FIGURE 8:**
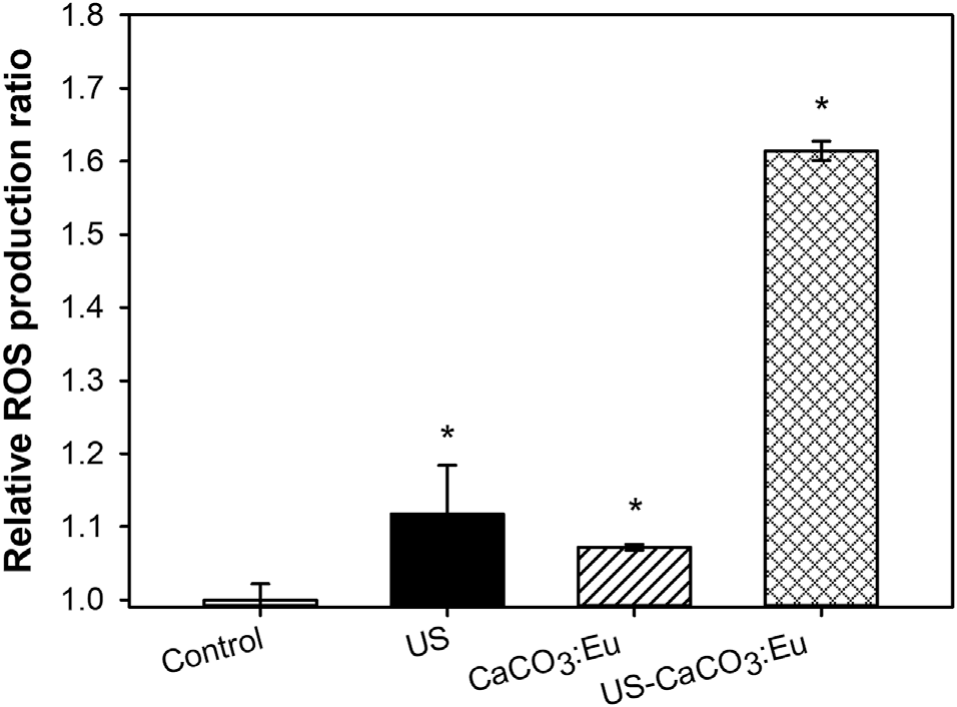
ROS production of 3T3-L1 cells treated with CaCO_3_: Eu under ultrasound irradiation, *p < 0.05.

Intracellular ROS production was measured by a staining kit of CM-H_2_DCFDA. The average fluorescence intensity of the control group was normalized as 1; the value of the other groups was normalized based on the intensity of control group as the relative value. The relative value would be in terms of the relative ROS production. After 3T3-L1 cells uptake the developed CaCO_3_: Eu and then exposed to ultrasonic irradiation, the relative ROS production of Control, US, CaCO_3_: Eu and US-CaCO_3_: Eu were 1.00 ± 0.02, 1.12 ± 0.67, 1.07 ± 0.01, and 1.61 ± 0.01, respectively, as shown in Figure 8. We could see that the 3T3-L1 treated separately only by ultrasound irradiation (US) and the CaCO_3_: Eu particles (CaCO_3_: Eu) would induce only small amount of ROS production; whereas the cells treated the combination of ultrasonic irradiation and the synthesized CaCO_3_: Eu (US-CaCO_3_: Eu) would induce great amount ROS generation.

From the results, we could tell that the developed CaCO_3_: Eu would be a good sonosensitizer to generate energy under the excitation of ultrasound irradiation to produce ROS for lipolysis application.

### The Efficacy of CaCO_3_: Eu Exposed to Ultrasound Stimulation to Induce Adipocyte Necrosis Under ROS Stress

The efficacy of CaCO_3_: Eu exposed to ultrasound stimulation to induce adipocyte necrosis under ROS stress was evaluated by WST-1 assay and live/dead stain to check the mitochondria activity and cell death rate, respectively.

**FIGURE 9:**
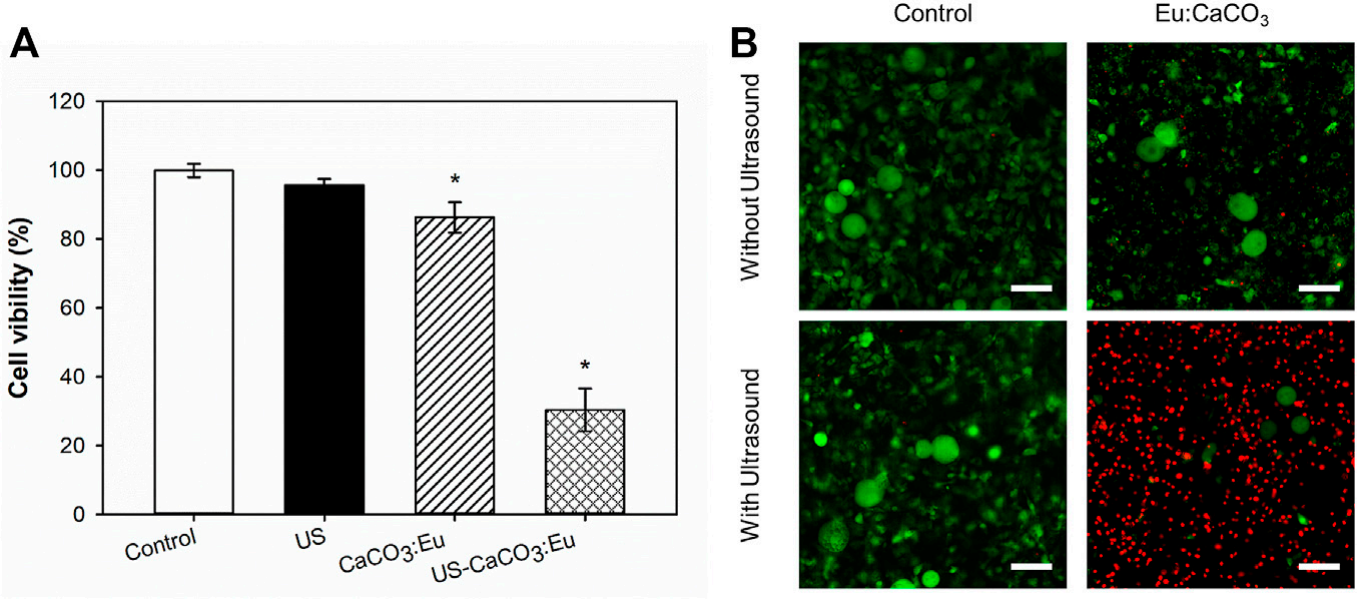
The cell viability of CaCO_3_: Eu exposed to ultrasound stimulation, evaluated by **(A)** WST-1 assay, *p < 0.05, and **(B)** Live/dead staining (scale bar: 100 μm).

The cell viability is the same as the previous description to normalize the OD value to the control group as 1; and then the value in the other groups was normalized referred to the control group to obtain a relative value. In Figure 9A, the adipocyte treated separately only by ultrasonic irradiation (US) and the developed CaCO_3_: Eu (CaCO_3_: Eu) would keep the mitochondria in normal function as control group (Control). In the contrary, the mitochondria function or cell viability was far less than that of the control group for the cells treated the combination of ultrasound irradiation and the developed CaCO_3_: Eu (US-CaCO_3_: Eu).

In the Figure 9B, the death rate of the adipocyte evaluated by live/dead stain had the same results as the previous WST-1 test; where the cell in green and in red were representative to living and dead cells, respectively. The results showed that the cells treated with the combination of ultrasound and developed particles had the highest death rate of 75%, compared with the control group.

From the results of WST-1 and live/dead stain, we believe that the cells treated with the combination of ultrasound and developed particles could effectively generate ROS to make the adipocyte toward necrosis under the stress.

### The Body Weight Growing Rate of the Rat Treated With CaCO_3_: Eu and Exposed to Ultrasonic Irradiation

**FIGURE 10:**
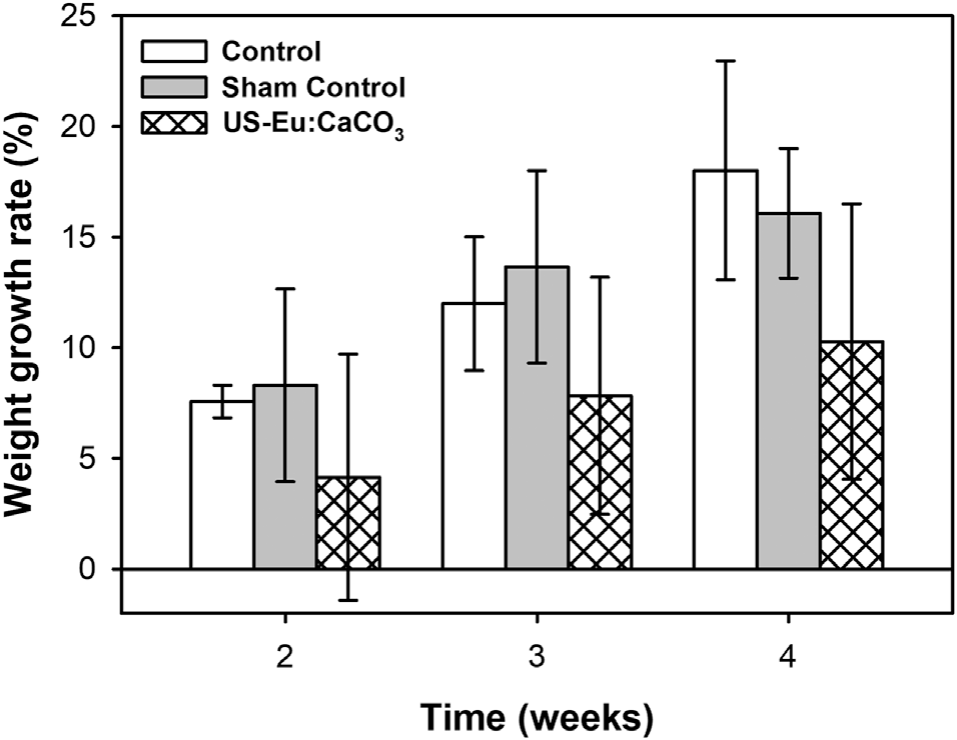
The weight growth rate of SD rats treated with CaCO_3_: Eu and exposed to ultrasonic irradiation.

The Figure 10 was the body weight growing rate of the rats injected with CaCO_3_: Eu to abdomen area and then applied with low-intensity ultrasound. The body weight growing rate of the rats without any treatment (Control) was much higher than the rats treated with the combination of CaCO_3_: Eu injection and low-intensity ultrasonic irradiation (US-CaCO_3_: Eu). The growth rate of control group was 7.57, 11.99, and 18.01 at week 2, 3, and 4, respectively. The growth rate of the group US- CaCO_3_: Eu was 4.16, 7.83, and 10.67, respectively, at week 2, 3, and 4.

### Waistline Measurement

**FIGURE 11:**
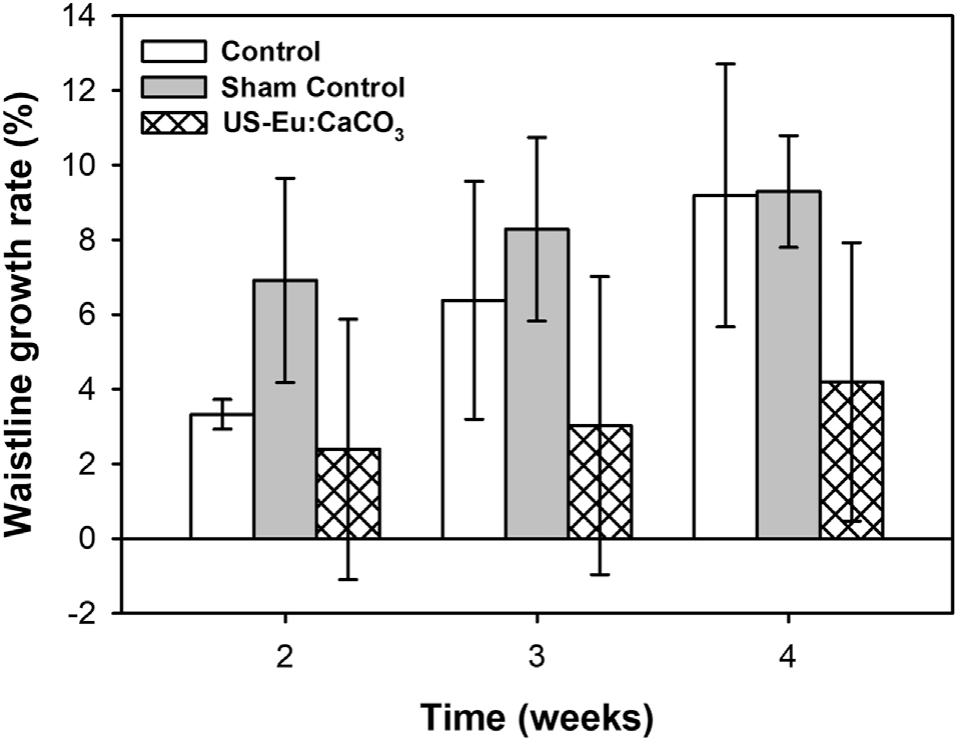
The growth rate of waistline in SD rats after treated with CaCO_3_: Eu injection and ultrasound irradiation.

Figure 11 was the waistline measurement of the experiment rats. The waistline of the rats treated with the combination of developed particle and low-power ultrasound was much lower than the control group and sham group. The waistline for the combination treatment was about 2.39, 3.02, and 4.19 at week 2, 3, and 4, respectively. The tendency was quite similar to the that of the body weight growth.

### The Growth Rate of Subcutaneous Fat

The growth rate of the subcutaneous fat was as shown in Supplementary Figure S1. The growth rate of the subcutaneous fat for the rats treated with the combination of the CaCO_3_: Eu injection and ultrasound stimulation at week 4 was 78.28%, compared with control group as 100%.

From the results of the experiment, the combination treatment could effectively inhibit growth rate on body weight, waistline, and subcutaneous fat.

## Discussion

CaCO_3_, comprises more than 4% of the mineral on earth’s crust and is found throughout the world. Its most common natural forms are chalk, limestone, and marble, produced by the sedimentation of the shells of small fossilized snails, shellfish, and coral over millions of years (Mar and Phyo, 2013; Castro-Alonso et al., 2019). CaCO_3_ has been widely used in medical applications, such as bone graft for tissue repair, biodegradable vehicle for drug and gene delivery etc. (Maleki et al., 2015; Song et al., 2018). In this study, we used Eu-doped calcium carbonate as sonodynamic reagent to combine with ultrasonic irradiation for body sculpture. Eu is a non-toxic rare earth element with an atomic number of 63, which belongs to the trivalent ion (Li et al., 2020). Eu could replace the calcium ion position of calcium carbonate to promote defects in calcium carbonate and increase the number of electron-hole pairs. Compared to divalent Ca ions, the doped Eu ions can obtain additional electrons, which creates a new energy level near the conduction band to reduce the energy gap effectively (Han et al., 2014; Wang et al., 2014). This makes the sonosensitizer more susceptible to ultrasonic irradiation and stimulates the generation of singlet oxygen and ROS in adipocytes for increasing the effective on lipolysis.

The CaCO_3_: Eu was successfully synthesized using the eco-friendly method. The crystal structure was identified by XRD, which was matched with the standard pattern of calcite CaCO_3_ (Figure 2). Zeta-sizer was used to analyze the particle size and distribution of the synthesized particles. The average particle size of CaCO_3_: Eu was 2.1 μm, which fall in the range of optimum particle size for cellular endocytosis (0.5–10 μm) (Hirota and Ter, 2012; Foroozandeh and Aziz, 2018). The particle size was further evaluated by TEM and SEM, those supposedly larger than that of Zeta-sizer due to the aggregation during the sample preparation before examined under electronic microscope. The real grain size was around 100–300 nm as shown in the electron-penetrated edge of the TEM picture (Figure 3A).

Sonosensitizers can be divided into organic-based compounds and inorganic-based particles (Rosenthal et al., 2004; Chen et al., 2014). The organic-based materials, such as porphyrin-based structures, were reported to have short life span under ultrasound irradiation and showed great cytotoxicity. The inorganic-based particles, such as Ag, Au, Pt, TiO_2_, and quantum dots, etc., have been used as sonosensitizer on sonodynamic therapy (SDT) for tumor/cancer treatment with better biostability and much longer life span (Xu et al., 2016). However, this kinds of material produce too much of ROS after exposed to ultrasonic stimulation, that is too strong and may result to the higher cytotoxicity (Serpe et al., 2012). In addition, the inorganic-based materials are not biodegradable in the human body. In the study, we develop a mild sonosensitizer CaCO_3_: Eu for body sculpture after exposed to ultrasound. It is a biodegradable particle that can be decomposed in endosome-lysosome complex, and then turn into carbon dioxide (CO_2_) and calcium ions (Ca^2+^), as described in the following series of reactions (Yang et al., 2019).

$${\text{CaCO}}_{3\left(s\right)}\to {\text{Ca}}^{2+}+{\text{CO}}_{3}^{2-}$$ (1)

$${\text{CO}}_{3}^{2-}+{\text{H}}^{+}\to {\text{HCO}}^{3-}$$ (2)

$${\text{HCO}}^{3-}+{\text{H}}^{+}\to {\text{H}}_{2}{\text{CO}}_{3}$$ (3)

$${\text{H}}_{2}{\text{CO}}_{3}\to {\text{H}}_{2}\text{O}+{\text{CO}}_{2}$$ (4)

where the CO_2_ could serve as bomb to break down the endosome-lysosome complex and as one of mechanisms to kill the adipocytes in the fat tissue. The high concentration of Ca^2+^ ions, decomposed from the CaCO_3_: Eu, could create a osmotic pressure to quickly escape from the complex environment. The adipose tissue with locally high level of Ca^2+^ ions would have the effect to the inhibition on the conversion of pre-adipocyte to adipocyte as following discussions.

Ca^2+^ ion has been investigated that was in association with adipocyte lipid metabolism, such as lipid synthesis and catabolism (Shapses et al., 2004; Duncan et al., 2007). Extracellular Ca^2+^ is also involved in the modulation of adipogenesis. It has been reported that high extracellular Ca^2+^ inhibits adipogenesis in 3T3-L1 pre-adipocytes (Jensen et al., 2004; Zhai et al., 2020). The process of pre-adipocyte differentiation of mature adipocytes is regulated by complex transcription factors, which can regulate the expression of hundreds of proteins responsible for establishing mature adipocyte phenotypes (Lowe et al., 2011). The two major adipogenic factors are peroxisome proliferator-activated receptor (PPARγ) and cytosine-cytosine-adenosine-adenosine-thymidine/enhancer-binding protein (C/EBP) (Farmer, 2006; Payne et al., 2009). Once the CaCO_3_: Eu is decomposed by cells, calcium ions diffuse into the interstitial space, turning the entire local environment into a high-calcium environment. A high-calcium concentration in the microenvironment activates the preadipocyte factor 1 (PREF1) expression, which causes the up-regulation of the transcription factor SOX9 (Wang and Sul, 2009), that could inhibit the formation of sterol regulatory element-binding protein (SREBP), C/EBP, and PPARγ for pre-adipogenic cell maturation (Jensen et al., 2004; Vergara et al., 2016; Das and Choudhuri, 2017; Pramme-Steinwachs et al., 2017). In this study, we cultured the cells with different concentration of calcium ions in the cell culture medium, this result was verified that, 3T3-L1 cells under high-calcium ion environment were inhibited the differentiation of fat precursor cells into adipocytes (Supplementary Figure S2).

Sonoluminescence is a sonosensitizer absorb energy from inertial cavitation followed bubble rapture after ultrasound applied to the local tissue to produce ROS. The ROS include superoxide ions (O_2_^−^), peroxide ions (O_2_^2−^), hydroxyl radicals (OH), and singlet oxygen (^1^O_2_), which can cause to cell death in fat tissue (Kuroki et al., 2007; Trendowski, 2014, 2015; Pang et al., 2016). The CMH_2_-DCFDA fluorescent dye was used to detect hydroxyl, peroxyl, and other ROS-active oxides in the cells. In this study, ROS production in the US group was 1.12 times higher than that in the control group. It is speculated that under the action of ultrasound, the generation of inertial cavitation finally causes the bubble to rupture, it could release strong energy that causes pyrolysis of surrounding water molecules, and producing hydroxyl groups in adipocytes. The production of ROS in the CaCO_3_: Eu group was not observed. In addition, compare with US-CaCO_3_: Eu and bare CaCO_3_ under ultrasound irradiation (US-CaCO_3_) group, ROS production of US-CaCO_3_: Eu was 1.24 times high than US-CaCO_3_ (Supplementary Figure S3). Meanwhile, in the US-CaCO_3_: Eu group, the ROS production is 1.61 times higher than control, which is presumed to be inertial cavitation and the generation of sonoluminescence, causing the acoustic-sensitive materials to be excited and produce singlet oxygen and superoxide. The results show that the combination of CaCO_3_: Eu and ultrasound treatment could produce more ROS free radicals on adipocytes. In addition, we also used the WST-1 and live/dead assays to verify the *in vitro* carving effect of CaCO_3_: Eu under ultrasound irradiation. The US group showed that only inertial cavitation acts on the pyrolysis of water molecules to produce hydroxyl, which has limited oxidative damage capacity in adipocyte. When ultrasound is applied to activate CaCO_3_: Eu, inertial cavitation pyrolysis produces hydroxyl and sonoluminescence excitation material, causing sonosensitizers to be excited to produce singlet oxygen and ROS. These results indicated that combination of CaCO_3_: Eu and ultrasound treatment could cause significant damage to the adipocyte.

The results of animal study did not remarkably change between the groups at the beginning. Nevertheless, at a specific time, the US-CaCO_3_: Eu sonodynamic treatment groups had a change in waistline within 4 weeks, and a statistical difference was reached in the fourth week. As the animal model used in this study was Sprague Dawley rats, the abdominal viscera and muscle tissue were removed, and the subcutaneous fat was measured based on the actual waist circumference. The results indicated the US-CaCO_3_: Eu on SD rats could significantly decrease the growth rate of body weight and waistline and reduce the storage of adipose tissue by the weight of subcutaneous fats. In addition, the reduction in subcutaneous fat cell volume was observed from fat tissue section between Control and US-CaCO_3_: Eu group (Supplementary Figure S4). Body temperature changes (Supplementary Figure S5), tissue sections (Supplementary Figure S6), and blood analysis (Supplementary Table S2) of the above animal experiments showed that the injection of acoustically sensitive materials in animals and the effects of ultrasound of the rats are safe, and does not affect the physiological condition and organs of the rats by the ultrasound effect. the CaCO3: Eu exposed to ultrasound irradiation on SD rats could significantly decrease body weight, waistline, and subcutaneous adipose tissue. In summary, the US-CaCO_3_: Eu sonodynamic treatment is demonstrated that has a great potential in the application of body sculpture.

## Conclusion

In the study, a sonosensitizer of Eu-doped CaCO_3_ was successfully synthesized to combine with low-intensity ultrasound for body sculpture. The results showed that the CaCO_3_: Eu had good biocompatibility and could produce ROS in adipocytes for lipolysis. In addition, the results showed that developed sonosensitizer could effectively inhibit the adipogenesis after treated with low-intensity ultrasound. After 4-weeks animal study, the developed CaCO_3_: Eu exposed to ultrasound irradiation on SD rats could significantly decrease the growth rate of body weight and waistline; and could reduce the storage of adipose tissue by the weight of subcutaneous fats. We could say that the combination of the developed Eu-doped CaCO_3_ and low-intensity ultrasound could effectively inhibit the adipogenesis without skin burning and charred sounding tissue; that would be a mild and non-invasive treatment for the body sculpture.

## Data Availability

The original contributions presented in the study are included in the article/Supplementary Material, further inquiries can be directed to the corresponding authors.
