# Supplementary material for: The Synthesis of Europium-Doped Calcium Carbonate by an Eco-Method as Free Radical Generator Under Low-Intensity Ultrasonic Irradiation for Body Sculpture
Source: Front Bioeng Biotechnol. 2021 Nov 19;9:765630. doi: 10.3389/fbioe.2021.765630 (PMC8639516; doi:10.3389/fbioe.2021.765630)
Supplement: Supplementary file 1 [file DataSheet1.docx]

Supplementary Material

# Supplementary Figures and Tables





**Supplementary Figure 1.** Subcutaneous fat percentage in SD rats induced by CaCO_3_:Eu activated by ultrasound.


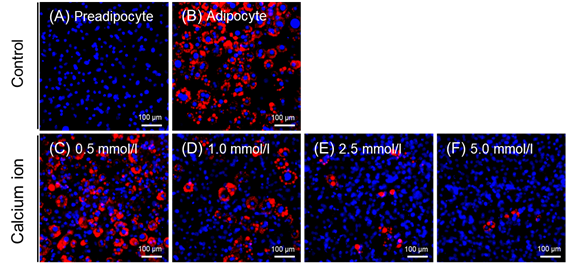


**Supplementary Figure 2.** Inhibition of differentiation of fat precursor cells into adipocytes and lipids accumulation under different calcium ion concentrations.





**Supplementary Figure 3.** ROS production of 3T3-L1 cells treated with US-CaCO_3_ and US-CaCO_3_:Eu, **p* < 0.05.

| **Control** | **US-CaCO_3_:Eu** |
| --- | --- |
| **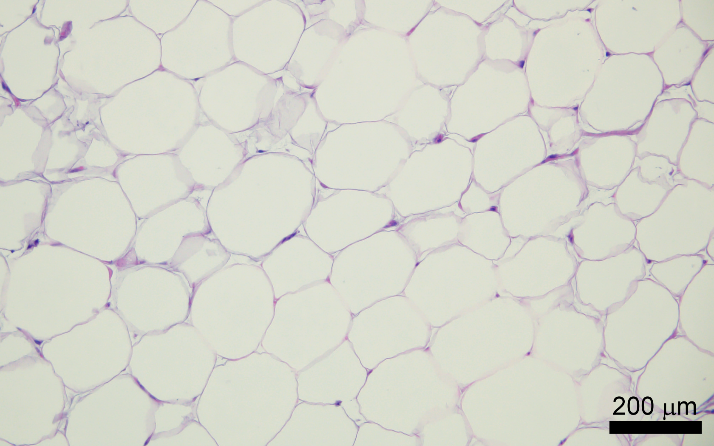** | **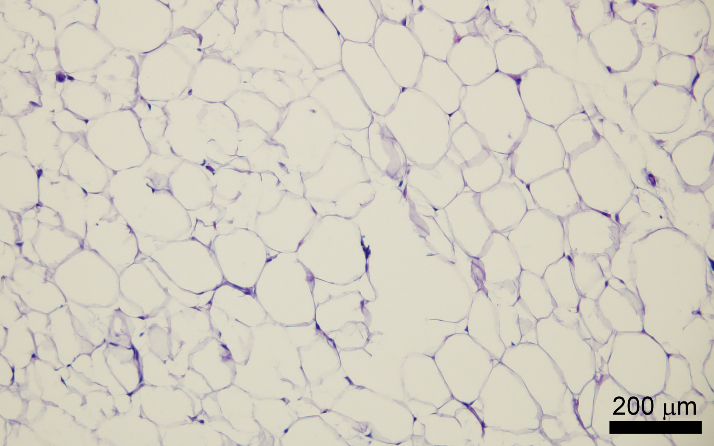** |

**Supplementary Figure 4.** Histological sectioning with H&E stain on subcutaneous fat tissue.





**Supplementary Figure 5.** Measurement of body temperature on SD rats.

**Supplementary Figure 6.** Histological sectioning with Hematoxylin and eosin (H&E) stain (scale bar: 100 μm).

**Supplementary Table 1.** Measurement of Eu concentration in CaCO_3_-Eu by ICP-MS.

| Sample | Eu (mg/g) |
| --- | --- |
| CaCO_3_ | ND |
| CaCO_3_:Eu | 112.5 |

**Supplementary Table 2.** Biochemical and hematological tests.

| Variables | NC | US-CaCO_3_:Eu | |
| --- | --- | --- | --- |
| WBC (K/μL) | 9.3 ± 2.9 | 7.3 ±2.6 | |
| NE (%) | 23.1 ± 8.8 | 18.1 ±10.6 | |
| LY (%) | 68.5 ±10.7 | 76.2 ±13.9 | |
| MO (%) | 4.9 ±0.5 | 4.0 ±1.6 | |
| EO (%) | 2.2 ±1.0 | 1.0 ±1.4 | |
| BA (%) | 1.4 ±0.39 | 0.7 ±1.1 | |
| RBC (M/μL) | 7.8 ±1.4 | 7.8 ±0.5 | |
| HGB (g/dL) | 15.66 ±0.6 | 13.9 ±1.8 | |
| HCT (%) | 49.4 ±7.8 | 43.8 ±3.8 | |
| PLT (K/μL) | 976 ±313 | 981 ±119 | |
| AST (U/L) | 121 ± 6 | 143 ±26 | |
| ALT (U/L) | 39.0 ±9.2 | 40.7 ±11.9 | |
| BUN (mg/dL) | 20.2 ±3.0 | 18.6 ±3.3 | |
| CRE (mg/dL) | 0.4 ±0.2 | 0.2 ±0.0 | |
| UA (mg/dL) | 1.5 ±0.2 | 1.2 ±0.5 | |
| TG (mg/dL) | 117 ±29 | 142 ±6 | |
| TC (mg/dL) | 111 ±1 | 73 ±12 | |
| Ca (mg/dL) | 11.0 ±0.5 | 9.3 ±0.6 | |
| WBC- White blood cell; NE- Neutrophil; LY- Lymphocyte; MO- Monocyte; EO- Eosinophil; BA- Basophil; RBC- Red blood cell; HGB- Hemoglobin; HCT- Hematocrit; PLT- Platelet; AST - Aspartate aminotransferase; ALT - Alanine aminotransferase; BUN - Blood urea nitrogen; CRE- Creatinine; UA- Uric acid; TG- Triglycerides; TC- Total cholesterol; Ca- Calcium. | | |  |
